# Interfacial Coupling Effect on Electron Transport in *MoS*_2_*/SrTiO*_3_ Heterostructure: An Ab-initio Study

**DOI:** 10.1038/s41598-017-18984-6

**Published:** 2018-01-15

**Authors:** Amreen Bano, N. K. Gaur

**Affiliations:** Department of Physics, Barkatullah University, Bhopal, 462026 India

## Abstract

A variety of theoretical and experimental works have reported several potential applications of *MoS*_2_ monolayer based heterostructures (HSs) such as light emitting diodes, photodetectors and field effect transistors etc. In the present work, we have theoretically performed as a model case study, *MoS*_2_ monolayer deposited over insulating *SrTiO*_3_ (001) to study the band alignment at *TiO*_2_ termination. The interfacial characteristics are found to be highly dependent on the interface termination. With an insulating oxide material, a significant band gap (0.85eV) is found in MoS_2_/TiO_2_ interface heterostructure (HS). A unique electronic band profile with an indirect band gap (0.67eV) is observed in *MoS*_2_ monolayer when confined in a cubic environment of *SrTiO*_3_ (STO). Adsorption analysis showed the chemisorption of *MoS*_2_ on the surface of STO substrate with *TiO*_2_ termination which is justified by the charge density calculations that shows the existence of covalent bonding at the interface. The fabrication of HS of such materials paves the path for developing the unprecedented 2D materials with exciting properties such as semiconducting devices, thermoelectric and optoelectronic applications.

## Introduction

The exfoliation of two-dimensional(2D) graphite commonly known as graphene has opened up a world wide research interest in atomically thin materials over the past decade^1^. A group of such materials in which interlayer interactions are governed by the Van der Waals(vdW) forces includes transition metal dichalcogenides (TMDs) which follows *MX*_2_ structure where M is a transition metal (Mo, W, Re, Nb etc.) and X is a chalcogen (S, Se,Te)^2^. These TMDs offers flexibility in tuning their electronic properties by reducing the number of layers from bulk to monolayer. It gives out from an indirect band gap in 3D systems to a direct semiconducting band gap in 2D monolayer^3,4^. Heterostructures (HSs) comprises of such 2D materials have provided a new realms in device application industry such as light emitting diodes^5^, photodetectors^6–8^ and field effect transistors^9–11^ due to their ability to align band between 2D planes^12–17^. It is relatively quite easy to fabricate a HS with two similer type of structures with lesser lattice mismatch. These heterostructure (HS) includes *MoS*_2_/*WS*_2_^18^, *MoS*_2_/*MoSe*_2_^19^, *MoS*_2_/*SnSe*_2_^20^, graphene/hBN^21–25^, *MoS*_2_/*WSe*_2_^26^. In the present work, we have used STO as a substrate which is an insulator with perovskite structure, widely used for the growth of multiferroics, colossol magneto-resistive manganites, high temperature superconducting cuprates^27^. Recently, a large number of exciting physical properties has been observed in various oxide HSs based on STO substrates such as charge writing^28^, resistance switching^29^, quasi-2D electron gas (q-2DEG)^30,31^, magnetism^32^, giant thermoelectric effect^33^ and colossal ionic conductivity^34^. These variety of functionalities offers potential applications in oxide electronics^35^, thermoelectric materials and solid oxide fuel cells (SOFCs)^36^. In 2004, Ohtomo and Hwang^30^ reported a high mobility electron gas at the interface between STO and *LaAlO*_3_ HS. Both the oxide materials are band insulators. The conductivity of the interface was found to be STO termination dependent i.e. for *TiO*_2_ terminated STO, the interface was conductive while for *SrO* termminated STO, it was insulating^31,37–43^.

Recently, several experimental investigations have been reported on *MoS*_2_/*TiO*_2_ interface HSs, but no theoretical attempts have been made. Motivated from this, we have studied theoretically the *MoS*_2_/*STO* HS with an *MoS*_2_/*TiO*_2_ interface using ab-initio approach. We have provided the computational details which are applied to probe several basic features of the electronic structure of *MoS*_2_/*TiO*_2_ interface in Section II. The results on the electronic band structure of HS and isolated sub-systems along with the chemisorption and chemical bonding occuring at the interface near Fermi level (*E*_*f*_) are reported and discussed in Section III.

## Computational Methods

**Figure 1:**
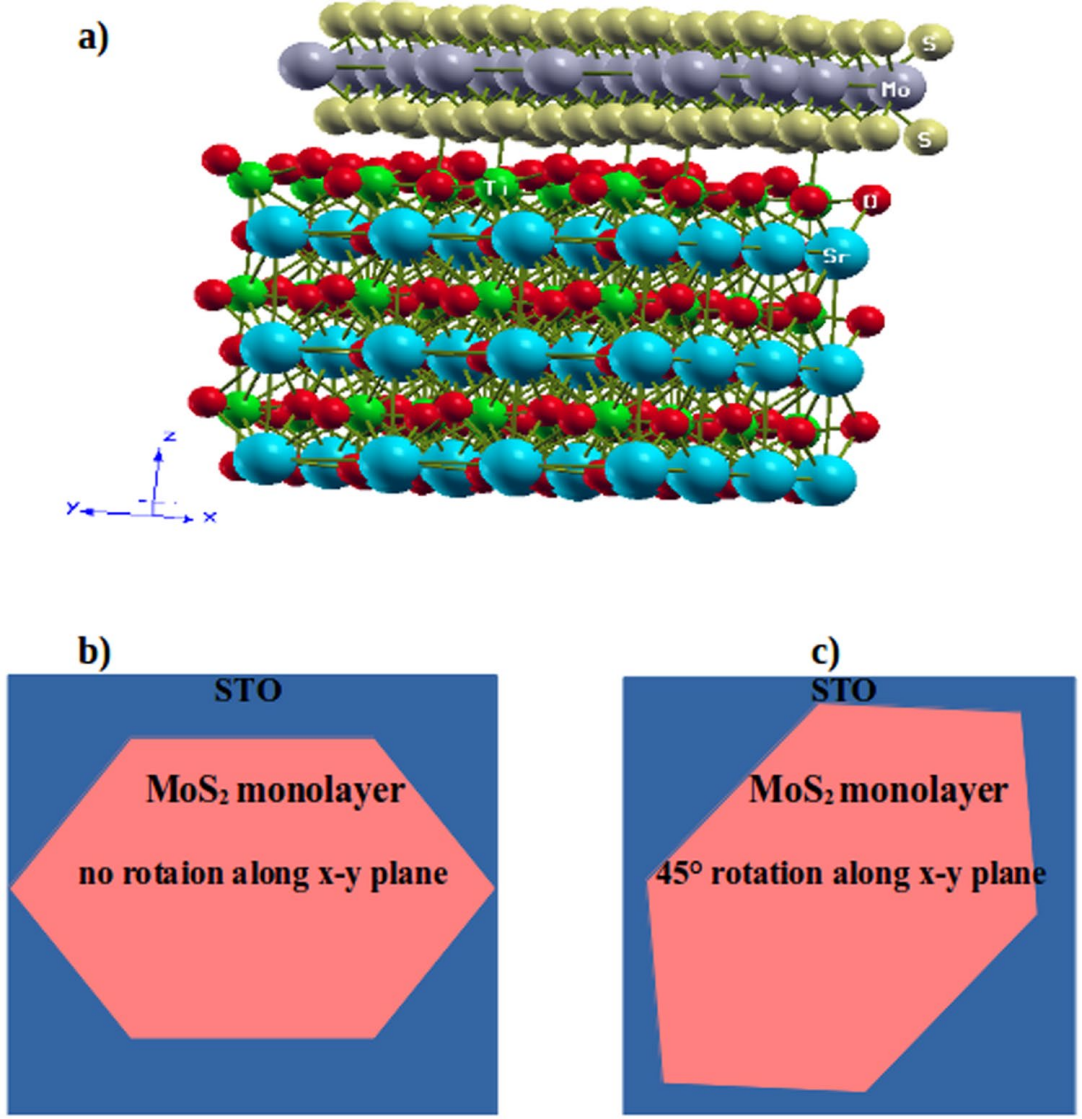
(**a**) Heterostructure of *TiO*_2_ terminated *MoS*_2_/*STO*; Gray balls indicates Mo atoms, Yellow balls shows S atoms, Blue balls shows Sr atoms, Green balls indicates Ti atoms and Red balls shows O atoms; diagramatic top view of HS showing (**b**) *MoS*_2_ monolayer over STO with no rotation and (**c**) 45° rotation along x-y plane.

To study the electron transport properties of *MoS*_2_/*STO* HS, density functional theory (DFT)^44^ calculations were carried out by Quantum Espresso Package^45^. The system is analysed by fully relaxing the HS with *TiO*_2_ terminated STO substrate containing supercell of size 2 × 2 × 1 and 4 × 4 × 1 *MoS*_2_ monolayer supercell as shown in Fig. 1(a). Perdew and Zunger functionals^46^ within local density approximations (LDA)^47^ is used to treat the electronic exchange-interaction. The input parameters, i.e. kinetic energy cutoff and K-mesh are fully optimized before proceeding to the calculations of electronic properties of the HS. Monkhorst-Pack^48^ 6 × 6 × 1 K-mesh for Brillouin zone integrations were employed with 544 eV of kinetic energy cutoff. We have taken the value of U = 4eV for effective Coulomb repulsion. All the calculations are based on projector-augmented waves (PAW)^49^ method. Generally, the PAW potentials are more accurate than ultrasoft pseudopotentials, because (1) Core radii of PAW potentials are smaller than the radii used for ultrasoft pseudopotentials, (2) PAW potentials reconstruct the exact valence wavefunction with all nodes in the core region. A vaccuum of 28 Å is provided normal to the interface in order to present the isolated slab boundary condition. Here, we have chosen a lateral lattice parameters of cubic STO a_0_ = 7.71 Å, which is optimized for freestanding STO. The lattice mismatch for the interface of HS is found to be 5%, which is expected as the crystal structures of both systems are different i.e. STO is cubic and *MoS*_2_ monolayer is hexagonal. The equilibrium interfacial distance (*d*_*eq*_) between *MoS*_2_ and surface of *TiO*_2_ terminated STO(001) is found to be *d*_*eq*_= 2.62 Å. This distance is obtained after relaxing the HS. We have studied the electronic band structures of the HS and *O* − 2*p* orbitals are observed at the Fermi level without crossing it with a significant band gap. In this work, we have carried out the spin-polarized calculation and our results indicates that *MoS*_2_/*STO* HS with *TiO*_2_ termination is nonmagnetic.

## Results and Discussion

### Electronic band structure

#### *MoS*_2_/*STO* Heterostructure

**Figure 2:**
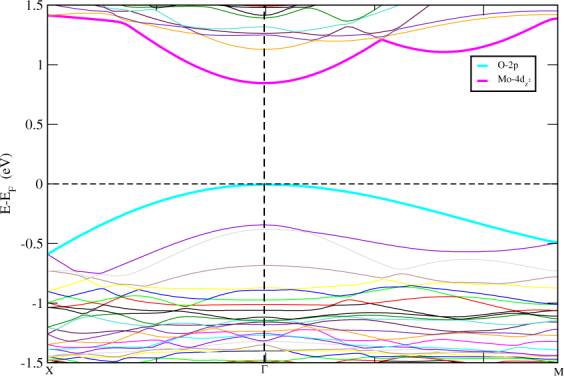
Electronic band structure of *TiO*_2_ terminated HS along high symmetry points X(0.5,0,0)-Γ(0,0,0)-M(0.5,0.5,0.0) and the horizontal dashed line implies the *E*_*f*_ set to zero energy at Y-axis.

We have studied the effect of *MoS*_2_ monolayer on the electronic properties of *TiO*_2_ terminated STO (001). The lattice constant of freestanding STO is 68% larger than that of *MoS*_2_ monolayer which could effect our results of the HS. Hence, we constructed a supercell consisting of a *TiO*_2_ terminated STO containing 2 × 2 × 1 substrate and 4 × 4 × 1 *MoS*_2_ monolayer. The remaining lattice mismatch is only about 5%, which is small enough and it does not effect the electronic structures in the interfaces. The fully relaxed atomic structure of the HS is shown in Fig. 1(a), where clear bonding at the interface can be seen between the two subsystems (STO and *MoS*_2_ monolayer). To make sure that the results we have obtained for the HS are correct, we rotated the *MoS*_2_ monolayer by 45° along x-y plane keeping the STO substrate fixed as shown in Fig. 1(b) and (c) and performed the calculations and found similer results as that of the HS with no rotation of *MoS*_2_ monolayer. To investigate the band alignment of *MoS*_2_/*TiO*_2_ interfaced HS, we have plotted the band structure of the HS. The band diagrams of the sub-systems of the HS are also studied to get a clear insight of the mechanism taking place in the complex HS. The band structure of *MoS*_2_/*TiO*_2_ interface HS is shown in Fig. 2. It can be seen that a band is sitting at the *E*_*f*_ at the valance band maximum (VBM) and energy gap (0.85eV) is visible in *TiO*_2_ terminated HS making it a semiconducting material. The band gap is found to be a direct gap at Γ. The effect of interfacial coupling in this HS gives exciting electronic nature as, an ideal monolayer of *MoS*_2_ is supposed to be a direct band gap material which is found at *K* high symmetry point while STO in bulk is an indirect band gap (M-Γ)^50^ insulating material. Here, when we couple these systems together, it gives out a semiconducting band gap of 0.85eV at Γ. This property brings the HS in the list of potential materials for optoelectronic devices. The conduction band minimum (CBM) is composed of Mo-4d electron states in Fig. 2.

**Figure 3:**
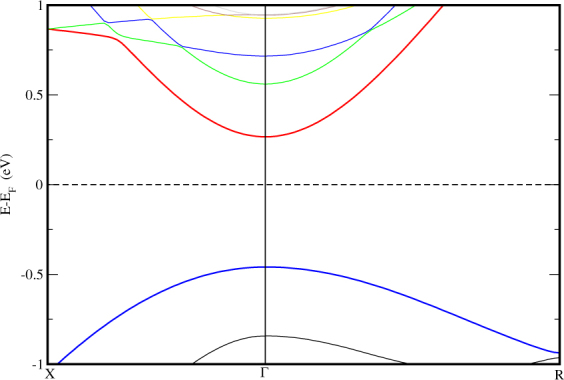
Electronic band structure of STO in *TiO*_2_ termination without *MoS*_2_. Horizontal dashed line implies the *E*_*f*_ set to zero energy at Y-axis.

Now we are interested to study the band structure of STO by removing the *MoS*_2_ monolayer. The electronic band structure of STO without *MoS*_2_ monolayer is shown in Fig. 3. We observed from Fig. 3 that when *MoS*_2_ is removed from *TiO*_2_ terminated HS, the VBM shifts down and CBM move towards *E*_*f*_ and reduced band gap (0.72eV). Hence, the effect of *MoS*_2_ monolayer on STO can be analysed as, when *MoS*_2_ monolayer is deposited on STO, a relatively large band gap is obtained and in place of Mo-4d states, now Ti-3d states are seen at CBM. The monolayer is influencing the energies of atoms of STO. Also, we found from Fig. 3 that, the direct band gap is not coming due to the monolayer but STO itself is giving out a direct semiconducting gap at Γ. Therefore, from Fig. 2, we conclude that *MoS*_2_/*TiO*_2_ interface is a semiconductor with a direct narrow energy gap. This indicates the effect of *STO* substrate with *TiO*_2_ termination on the electronic properties of *MOS*_2_. It shows that *STO* substrate can be used as an ideal substrate for *MoS*_2_-based device applications. Band gaps obtained for other HSs based on *MoS*_2_ are enlisted in Table 1.

**Table 1:** Band gap (*E*_*g*_) of other heterostructures based on *MoS*_2_.

Structures	Reported Band gaps (eV)
*MoS*_2_/*WS*_2_	1.25^54^
*Stanene*/*MoS*_2_	0.067^55^
*MoS*_2_/*SiC*	1.34^56^
*ZnO*/*MoS*_2_	1.48^57^
*Graphene*/*ZnO*/*MoS*_2_	1.01^57^

#### Subsystems-STO and *MoS*_2_ Monolayer

**Figure 4:**
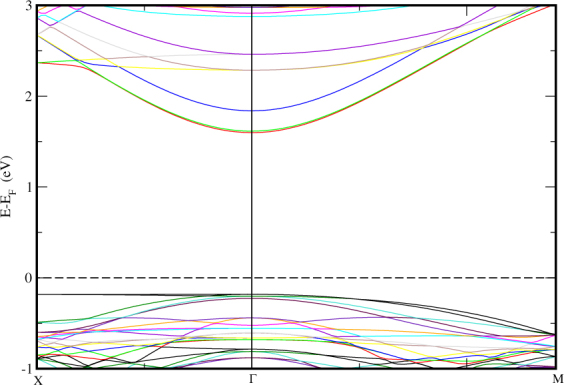
Electronic band structure of Cubic STO with vacuum along c-axis. High symmetry points are X(0.5,0,0)-Γ(0,0,0)-M(0.5,0.5,0.0) and the horizontal dashed line implies the *E*_*f*_ set to zero energy at Y-axis.

**Figure 5:**
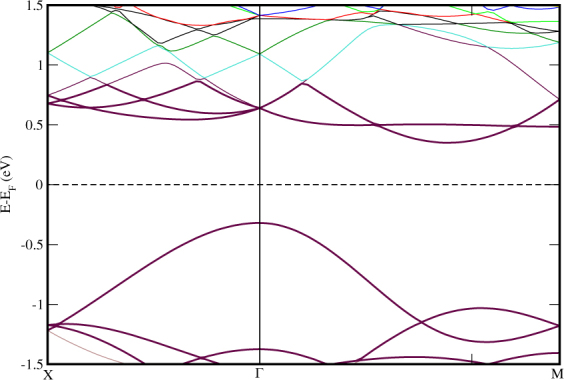
Electronic band structure of *MoS*_2_ Monolayer with cubic confinement. Horizontal dashed line implies the *E*_*f*_ set to zero energy at Y-axis.

We have also studied the band structure of STO and *MoS*_2_ monolayer confined within the structural parameters of the STO (001) (2 × 2 × 1) seperately in order to have a comparative study. We can see in Fig. 4, the ideal cubic STO substrate appears to have a direct band gap (at Γ) of 1.77 eV which is reduced from the bulk STO band gap (3.2eV) arising from *p*_*x*_, *p*_*y*_ orbitals^51^ on the surface layer O ion whose on-site energy is raised by the absence of interations along c-axis. In Fig. 5, the band structure of *MoS*_2_ monolayer is shown. Here, we find an unexpected and unique character of monolayer confined in a cubic unit cell of STO, i.e. it gives out an indirect band gap of 0.67eV. Hence, we can say that the direct band gap of the HS is obtained only due to the contribution of STO with *TiO*_2_ termination. We know that, Silicon is an indirect band gap (1.1 eV) semiconductor (X-L). This reduces its efficiency in electron transport. Hence, we propose a hypothetical view based on our results of monolayer in Fig. 5. If we could fabricate stable freestanding silicon monolayer in honeycomb arrangement as that of ideal *MoS*_2_ monolayer, we may get a direct band gap which is obtained in case of ideal *MoS*_2_ monolayer. An ideal monolayer of *MoS*_2_ gives a direct band gap in honeycomb arrangement, when we confined it in cubic STO dimensions, it becomes an indirect band gap material. In the same way, if the cubic Si could be grown as honeycomb monolayer of *MoS*_2_, it might become a direct band gap system. This hypothesis can be tested by experimental efforts. The *E*_*f*_ of STO alone lies at 0.86 eV whereas for *TiO*_2_ terminated HS, it is found at 1.9893 eV, i.e. *E*_*f*_ of HS lies approximately 1eV above STO valance band maximum (VBM), which nearly at the middle of STO substrate energy gap. Thus, even if strong bonding takes place at the interface, the electronic states at the *E*_*f*_ would deteriorate swiftly into STO layer, this supports the use of thin STO film as substrate^52^.

### Partial Density of states

**Figure 6:**
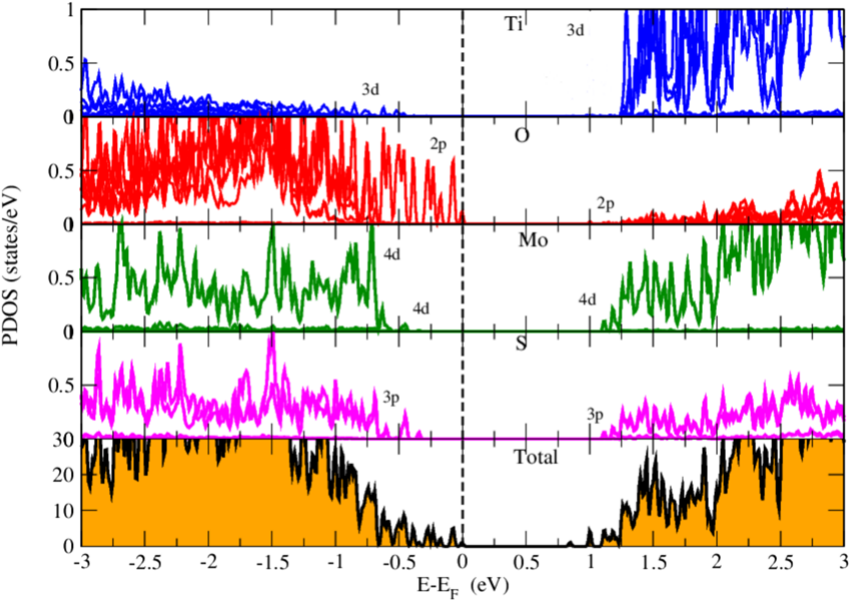
Partial density of states of *TiO*_2_ terminated HS. The *E*_*f*_ is set to zero.

The partial density of states (PDoS) further elucidate the electronic band structure. We have displayed the PDoS of *TiO*_2_-terminated HS in Fig. 6. We have observed that the band we saw in Fig. 2, sitting at the *E*_*f*_ is the O-2p states coming from the surface layer of STO. The VBM of HS is composed of O-2p states while CBM is found at 0.85eV with mixed states of Ti-3d, Mo-4d and S-3p. Ti-3d states are present in VB with relatively low intensity PDoS peaks i.e. contribution of Ti in VB is very feeble. Whereas, Mo-4d and S-3p states are clearly seen in both sides of *E*_*f*_. Also, O-2p states are contributing in VB region actively and are found at *E*_*f*_ but not crossing it. Low intensity peaks of O-2p states are seen is energy below 1.25 eV in CB region.

**Figure 7:**
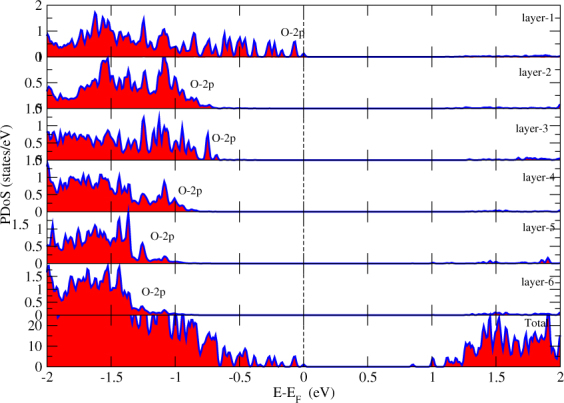
Partial density of states of O atom of each layer in *TiO*_2_ terminated HS. The *E*_*f*_ is set to zero.

We studied the contribution of O atoms of each layer of STO to find that O atom of which layer of STO is observed at *E*_*f*_. Fig. 7 shows the O-2p states of each layer of STO. We begin our analysis from the bottom layer of STO. The common character of O-2p states in each layer is that their contribution in CB is almost negligible. The O-2p states of bottom layer of STO are seen in VB from −1.1eV to far energy range. They are absent near *E*_*f*_. As we move to the surface layer of STO, the contribution of O-2p states are getting more close to *E*_*f*_ and at the surface layer, O-2p states are found exactly at the *E*_*f*_ but they are not crossing it. These surface O atoms with Mo-4d states of monolayer are giving rise to the existence of energy gap as maximum of VB is composed of O-2p states and minimum of CB is composed of Mo-4d states.

### Chemical Bonding

**Figure 8:**
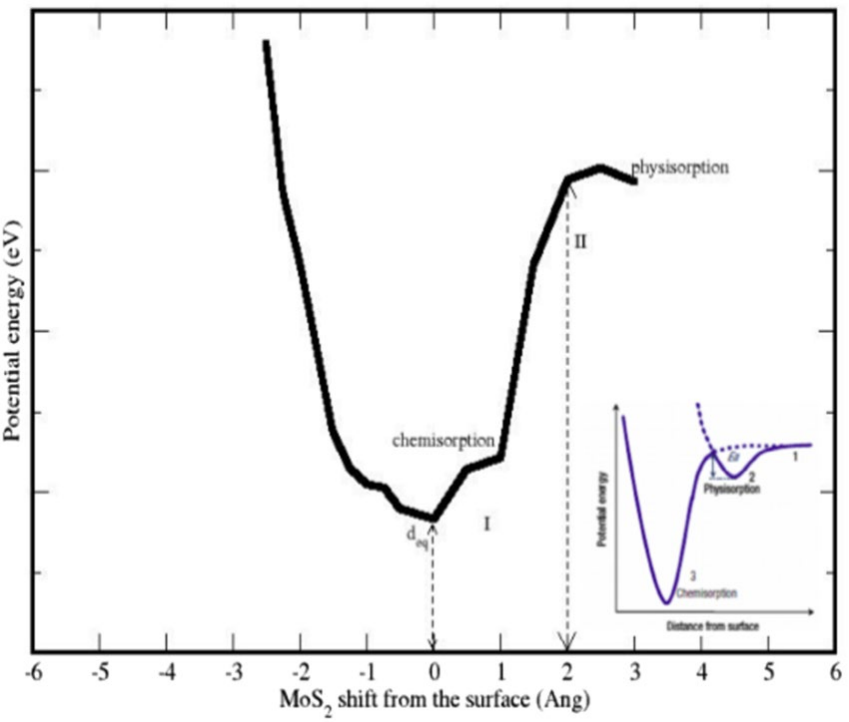
Nature of adsorption of *MoS*_2_ on the surface of STO (001); *MoS*_2_ monolayer is shifted in units of Å. *d*_*eq*_ is taken as 0 shift and further shifts are added to *d*_*eq*_; figure in inset shows the ideal adsorption curve shows the trend of chemisorption and physisorption^53^.

In further calculations, we have investigated the nature of adsorption of *MoS*_2_ on substrate STO, i.e., whether it is chemisorbed or physisorbed. In chemisorption, chemical reaction occurs between the surface of subtrate and the adsorbate (*MoS*_2_) and hence, the new chemical bonds are generated at the interface. Whereas, in case of physisorption, also called as physical adsorption, the electronic properties of the adsorbate are barely perturbed upon adsorption. The bonds can be seen at the interface between the substrate and the adsorbate in Fig. 1(a). This motivates us to investigate the nature of adsorption of the *MoS*_2_ monolayer. In general, vdW interactions exists at the interface of honeycomb like HSs, but in this case, the lattice arrangement is having the cubic STO dimensions hence it is considered that the interactions at the interface will no longer be weak vdW interactions. In order to analyse the nature of adsorption, we randomly shifted the *MoS*_2_ monolayer from the equilibrium distance at the interface *d*_*eq*_ where the *MoS*_2_/*TiO*_2_ interface distance is 2.62 Å, upwards upto 5.62 Å and downwards upto 0.3 Å normal to the surface of *TiO*_2_ terminated STO substrate. We have shown the results in Fig. 8. Here we can see two regions I and II. Region I shows the adsorption charateristics of *MoS*_2_ monolayer when the distance from surface of substrate ranges from 2.62 Å to 3.80 Å. We observed an increament in the potential energy of the HS in this region. A deep valley is obtained at *d*_*eq*_ which provides the traces of the chemisorption of adsorbate (*MoS*_2_) over surface of *TiO*_2_ terminated STO substrate. As the distance at the interface is further increased, the potential energy starts to fall down. This decreament is recorded in the range from 3.8 Å to 5.62 Å, as we have studied this response in range of distance 0.3 Å, upwards upto 5.62 Å only. As we further decreased the distance lesser than *d*_*eq*_, we can clearly see an abrupt increase in potential energy which indicates the presence of electronic repulsion. If we further decrease this distance, the energy will tend to infinity under the effect of nuclear repulsion. From Fig. 6, we found that the *MoS*_2_/*TiO*_2_ interface is a semiconductor with covalent bonding occuring between Mo-4d and O-2p states. This hybridization of electronic states makes our statement on chemical adsorption more stronger.

**Figure 9:**
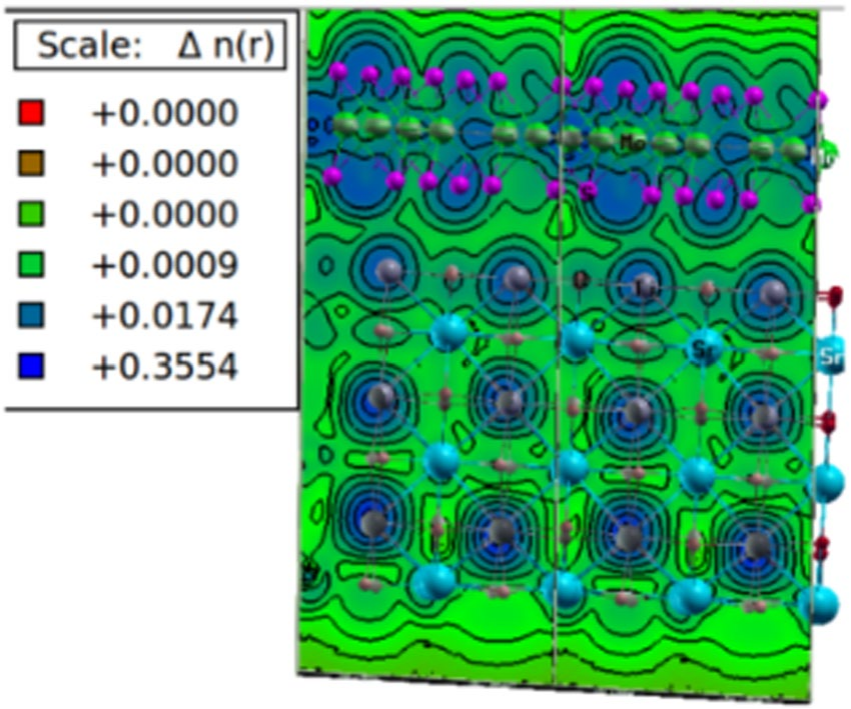
Charge density of *TiO*_2_ terminated HS in (010) plane. The color plate is at right side showing the charge transfer and bonding character at the interface. Atoms are presented as; Blue balls- Sr, Gray balls- Ti, Red balls- O, Magenta balls- S and Green balls- Mo.

The presence of chemisorption of *MoS*_2_ monolayer over STO surface can be justified by inspecting the nature of chemical bonding between the atoms at the interface. To investigate the bonding characteristics between atoms at the interface, we performed the charge density calculations. Charge density provides the information about bonding/repulsion between *MoS*_2_ and STO. Fig. 9 shows the charge density in (010) plane. Maximum values implies the regions where charge increases when *MoS*_2_ and STO coupling takes place. In the substrate STO, we can see the isolated closed spherical isolines around the bottom atoms. These closed isolines indicates the existence of ionic bonding between the atoms of STO at the bottom layer. As we move upwards, ionic bonding is still present till the surface layer of STO. A clear covalent bonding between the surface atoms of STO and *MoS*_2_ monolayer can be seen at the interface. It is considerd that if ionic bonding exists at the interface, the material should be a metal and if there exists an energy gap, it suggest a covalent bond between the atoms. Thus the presence of semiconducting band gap in HS (0.85eV) justifies our results of existance of covalent bonding obtained from adsorption analysis and charge density calculations. This bonding is arising due to the hybridization of O-2p and Mo-4d states in VB region which can be seen from Fig. 7 where the 2p energy states of O and 4d and 3p states of Mo and S atoms respectively are present in the energy range −0.3eV to far VB region. This mixing of states results in the strong covalent bonds between O-2p and Mo-4d states with small contribution of S-3p states.

## Conclusion

In summary, we have investigated the electronic band structure and chemical bonding of the *MoS*_2_ monolayer deposited over the STO substrate with *TiO*_2_ termination within the framework of density functional theory. In order to authenticate our results, we have performed all the calculations for the HS with 45° rotated *MOS*_2_ monolayer along x-y plane keeping the substrate fixed. We obtained similar results for such arrangement and hence validates our findings. The interfacial distance (*d*_*eq*_) between *MoS*_2_ and surface of *TiO*_2_ terminated STO(001) is found to be 2.62 Å. The conduction band is mainly composed of Mo-4d and S-3p states along with small contribution of Ti-3d states as well, while valance band is containing O-2p states near *E*_*f*_ which is playing the major roll in the semiconducting nature of the HS. Surface O atoms of STO substrate are found at *E*_*f*_. A semiconducting band gap is found (0.85eV) and *MoS*_2_ is strongly bonded with the substrate which is seen in the chemical bonding analysis. A unique nature of isolated *MOS*_2_ monolayer confined in STO lattice parameters is observed i.e. it gives an indirect band gap of 0.67eV. This finding can be examined experimentally for application purpose in the case of Si which could be a breakthrough in semiconducting devices. Our adsorption analysis suggests the chemisorption of *MoS*_2_ on surface of STO substrate with *TiO*_2_ termination. *MoS*_2_/*TiO*_2_ terminated HS with a semiconducting band gap offers a potential candidature in thermoelectric and optoelectronic applications.
